# Turning benign envy into engagement: the moderating role of inclusive leadership in nursing

**DOI:** 10.1186/s12912-025-03999-6

**Published:** 2025-11-17

**Authors:** Chunjie Lv, Muna I. Alyousef, Sajid Rahman Khattak, Usha Moorthy, Husain Mohammed Al Hakami, Fawziah B. Alharthi, Gul Erkol Bayram, Sami Znaidia, Sathishkumar Veerappampalayam Easwaramoorthy, Juan F. Espinosa-Cristia

**Affiliations:** 1 Renmin University of China, Beijing, 100872 China; 2 Department of Management Information System, College of Business Administration, University of Hail, Hail City, 81451 Saudi Arabia; 3 Department of Tourism Guide, Sinop University, Sinop City, Türkiye; 4 Department of Information Technology, Manipal Institute of Technology Bengaluru, Manipal Academy of Higher Education, Manipal, Bengaluru City, Karnataka India; 5 Department of Social Studies, College of Humanities and Social Sciences, King Saud University, Riyadh City, Saudi Arabia; 6 Department of Management, College of Business Administration, Taif University, Taif City, Saudi Arabia; 7 Laboratoire de Recherche (LR 18ES19), Synthese Asymetrique et Ingenierie Moleculaire de Materiaux Organiques pour l’Electroniques Organiques, Faculte des Sciences de Monastir, Universite de Monastir, Monastir City, 5000 Tunisia; 8 School of Engineering and Technology, Sunway University, No. 5, Jalan Universiti, Bandar Sunway, Sunway City, Selangor Darul Ehsan 47500 Malaysia; 9 Escuela de Ingeniería Comercial, Universidad Técnica Federico Santa María, Valparaíso City, Chile

**Keywords:** Benign envy, Help-seeking behavior, Learning behavior, Inclusive leadership, Work engagement

## Abstract

**Purpose:**

This study examines the constructive role of benign professional envy in predicting nurses’ work engagement, highlighting the sequential mediating effects of help-seeking and learning behaviors, as well as the moderating influence of inclusive leadership.

**Design/methodology/approach:**

Data were collected from nurses working in Turkish hospitals using a questionnaire. Using structural equation modelling (SEM), this study tested a moderated sequential mediation model grounded in the Conservation of Resources (COR) theory.

**Findings:**

The results revealed that benign professional envy positively and significantly predicted nurse work engagement. Furthermore, help-seeking and learning behaviors sequentially mediate this relationship, indicating that envy motivates nurses to seek advice and engage in active learning, which, in turn, enhances engagement. Importantly, inclusive leadership significantly moderates the indirect effects, such that the positive impact of benign envy on engagement through help-seeking and learning is stronger under conditions of high-inclusiveness leadership.

**Practical implications:**

The findings suggest that healthcare managers and nurse leaders should reframe benign envy as a potential resource, rather than a liability. By fostering inclusive leadership practices and encouraging help-seeking and learning behaviors, hospitals can transform envy into a driver of engagement, ultimately improving nurses’ well-being, reducing turnover, and enhancing patient care outcomes.

**Theoretical implications:**

This study extends COR theory by demonstrating how benign envy initiates a gain spiral of resources when supported by adaptive behavior and inclusive leadership. It also contributes to the literature on emotions in nursing by clarifying the mechanisms through which envy fosters engagement in high-demand healthcare settings.

**Originality/value:**

This study is among the first to investigate the constructive dynamics of benign envy in the nursing profession, offering a novel model that integrates emotional, behavioral, and leadership perspectives to explain work engagement.

**Clinical trial number:**

Not applicable.

## Introduction

Nurses are usually considered the pillar of healthcare systems, playing a crucial role in ensuring health service delivery, patient safety, service quality, and overall system resilience. The novel coronavirus disease (COVID-19) pandemic has further emphasized the essential contributions of nurses, while concurrently exposing them to extreme emotional stress, resource restraints, and heavy workloads [1, 2]. Moreover, in the recent era, factors such as healthcare workforce shortages, rapid technological development, and dynamic patient needs have made it indispensable for nurses to engage in continuous learning and development not only to sustain robust professional capabilities but also to offer better patient care services (World Health Organization, 2020). Therefore, understanding the psychological and motivational factors that uphold their commitment and professional development has become increasingly essential. Among these factors, benign professional envy (BPE) has emerged as a stimulating yet underexplored emotional concept [3]. Unlike malicious envy, which is characterized by antagonism and aggression, benign envy inspires employees to contend with and learn from more successful peers [4].

In the context of Turkish health services, the demand for qualified, committed, and highly engaged staff is generally persistent due to systematic reforms, brain drains of skilled workers, and dynamic public expectations [5]. Prior research related to nurses, particularly in the Turkish context, explored factors such as turnover intention [6, 7], burnout [7], job satisfaction [7], work-family conflict [8], workplace incivility [9, 10], intention to quit [11], and organizational commitment [12]. However, limited attention has been paid to emotional and social dynamics, such as professional envy which may help shape nurses attitudes and behaviors in the workplace.

Professional envy is frequently viewed as a negative emotion activated by upward social comparisons and is usually linked with flawed outcomes such as turnover intentions, job dissatisfaction, and emotional exhaustion [13]. However, a recent investigation recommends a more novel perspective: when managed usefully, envy can provoke individuals to attain important resources by imitating esteemed peers, pursuing feedback, or investing in learning efforts [14]. This conceptualization aligns with benign envy, which augments goal motivation and self-development rather than resentment or disruption [15]. Grounded in this positive perspective, we suggest that BPE among nurses can encourage help-seeking behavior which refers to a proactive strategy to gain knowledge and resources from others [16]. Consequently, help-seeking may stimulate learning behavior, resulting in subsidizing continued work engagement, an anticipated outcome in healthcare settings. These sequential mediating conduits reflect the central proposition of the conservation of resources theory that resource acquisition nurtures further resource gains, resulting in adaptive outcomes. In addition, it has been noticed that the effects of PBE are not identical across organizational settings [10, 13]. Certain contextual factors, such as leadership, can affect these relationships. Inclusive leadership, characterized by openness, accessibility, and support for varied viewpoints, may shape how nurses understand and react to feelings of envy [17, 18]. Such leaders can shape a psychologically safe work atmosphere where help-seeking is stimulated rather than defamed, thus augmenting the positive transformation of envy into productive behavior [19, 20].

Although these paradigms are notably important in both theoretical and practical realms, a substantial research gap remains concerning the positive potential of BPE, particularly in the Turkish nursing context. Most previous studies have focused on the detrimental consequences of envy, such as turnover intentions [21], job insecurity [22], intention to quit [23], and emotional exhaustion [24], while overlooking how benign envy can act as a motivational force for constructive behavioral outcomes. However, few studies have examined the positive consequences of professional envy in the workplace, such as innovative work behavior [25], employee performance [26], job engagement [27], and self-efficacy [3]. In addition, the literature has devoted limited attention to the underlying mechanisms through which benign envy functions to stimulate professional improvement and emotional commitment at work, particularly under contextual stimuli such as inclusive leadership. Thus, this study attempts to bridge these gaps by offering a more comprehensive and theory-driven framework.

Thus, this study offers several notable contributions to nursing professional development and well-being. First, this study extends the psychological dynamics literature by shifting the emphasis from the harmful facets of envy to its potential to encourage productive outcomes, such as work engagement. Second, the most important contribution of this study is the introduction and empirical validation of the unique chain mediation mechanism, where BPE improves nurses’ help-seeking behavior, which subsequently promotes learning behavior and, in the end, advances work engagement. Third, adding contextual enablers (e.g. inclusive leadership) to the model is another vital contribution of the present study. By investigating the moderating role of inclusive leadership, this study offers insights into how inclusive and helpful work settings can magnify the productive effects of envy on resource gain processes. Fourth, the application of the Conservation of Resources (COR) theory [28] in business organizations is common; however, its application in the nursing context, particularly by elucidating how envy can act as a motivational resource that initiates a gain spiral, leading to further personal and professional resource development, is rare and novel. Together, these contributions extend our understanding of how emotions, social factors, and contextual resources are interrelated to produce adaptive outcomes in resource-strained clinical settings.

## Literature review and hypothesis development

### Benign professional envy and nurse work engagement

Traditionally, professional envy has been described as a negative workplace emotion, referring to a yearning triggered by or dissatisfaction with another’s achievements, superior qualities, resources, or benefits in the same professional setting [29]. In nursing contexts, where appreciation, competence, proficiency, and access to career prospects are highly perceptible, envy may arise when nurses compare themselves to effective peers. While envy is usually connected with dysfunctional work behaviors [30], evolving research has explored its constructive potential, specifically when employees respond to envy by rivalling esteemed coworkers and endeavoring for self-development [31].

In contrast, work engagement refers to a “positive work-related state categorized by vigor, dedication and absorption” [32, p. 72], and is crucial in clinical settings. Goyal and Kaur [33] noted that engaged nurses have higher retention ratio, reveal greater quality care, lower intention to quit, and higher patient satisfaction. Notably, engagement signifies a state that entails important personal and psychological resources; therefore, understanding its precursors is crucial to addressing employees’ challenges in healthcare.

From a COR perspective [28], in the work setting, employees struggle to keep, acquire, and nurture resources (respect, professional capability, and self-confidence). Professional envy can serve as a precursor that stimulates employees to reevaluate their resource standing and identify perceived resource gaps between themselves and others. However, Gan [13] noted that in scenarios where nurses have adequate personal and contextual resources (e.g. supportive climate, resilience, flexibility, and inclusive leadership), envy can contribute to resource attainment actions, such as skill acquisition, increased effort, and learning, ultimately leading to nurturing engagement. Prior research also supports this notion; for instance, BPE can encourage ambitious behavior, motivating employees to improve task performance and engage in innovative work behavior [25, 26]. In the nursing context, such envy can inspire nurses to participate in more challenging tasks, pursue additional certifications, and improve their clinical skills. These purpose-driven behaviors can culminate in improved commitment, as nurses feel more experienced, skilful, accepted, and psychologically engaged in their clinical jobs [34].

Similarly, Liu et al. [35] found that competitive yet supportive environments permit envious employees to direct their emotions toward positive outcomes, such as innovation and engagement. Lee and Duffy [36] showed that envy can enhance job performance when mediated by goal pursuit. These findings not only support the notion that envy always leads to maladaptive behaviors but also support the COR suggestion that nurses feeling envy may participate in resource-building activities to close the resource gap, thus instigating the gain spiral [37]. Hence, these findings suggest that BPE, particularly under supportive conditions, can act as a motivational resource, encouraging nurses to participate more intensely in their task performance. By struggling to contest or beat their peers, they put more effort and dedication into their jobs, ultimately leading to increased work engagement. Therefore, we propose the following hypothesis:

#### H1

Benign professional envy is positively and significantly related to work engagement.

### Benign professional envy and help seeking behavior

Recent empirical and theoretical investigations recommend that benign professional envy – an enticing emotion activated by upward social comparisons–may act as a productive antecedent of career development in high-demand occupations [27, 38]. In nursing, where constant learning and proactive coping are vital, benign envy may arise when nurses recognize peers with higher recognition, clinical skills, and emotional flexibility. According to the COR viewpoint [28], this experience can be theorized as a perceived resource inconsistency, stimulating energies to preclude resource loss and pledge resource gains. COR posits that individuals are determined to safeguard prevailing resources and procure new ones when confronted with intimidation or discrepancies. Therefore, BPE may not indicate faintness but rather an acknowledgement of developmental opportunity, inspiring nurses to engage in preemptive behaviors such as help-seeking [39, 40].

Help-seeking refers to “actively importuning support, knowledge, or guidance from others” [41, p. 2] and is a crucial yet underutilized coping strategy in nursing due to humiliation, stigma, fear of judgment, and hierarchical structures. However, when BPE are assessed as self-referential but non-threatening stimuli, emotional hurdles to help-seeking decrease by reevaluating or rethinking more experienced peers as counsellors rather than opponents [38]. This is consistent with the COR perspective of resource cavalcades, in which the attainment of one resource (e.g. social support through help-seeking) enables the gain of others (e.g. self-efficacy and skill improvement). Therefore, we propose that BPE functions not as a maladaptive behavior but as a motivational antecedent to resource-enriching behaviors and plays a crucial role in nursing resilience and career development. Thus, we propose the following hypothesis:

#### H2

Benign professional envy is positively and significantly related to nurses’ help-seeking behavior.

### Help seeking behavior and learning behavior

Help-seeking behavior has been widely acknowledged as a preemptive and self-regulatory mechanism that stimulates individual development and workplace learning [42]. In the present context, help-seeking is a deliberate effort to gain knowledge, skills, and guidance from others. In addition, help-seeking is not simply a sign of dependency but a thoughtful action to access external resources [43]. In healthcare contexts, nurses’ help-seeking behavior is crucial for acquiring novel skills, adapting to dynamic challenges, and solving task-related ambiguity. This is consistent with the COR tenets that individuals are motivated to obtain, protect, and build resources, particularly when they recognize fears of their present resources or perceive an opportunity for expansion [28]. In the present context, help-seeking acts as a strategy to recompense for resource paucities (e.g. lack of skill or confidence) or to reinforce resource pools (e.g. expertise, support networks).

Moreover, in professions characterized by a severe shortage of workers, such as nursing, seeking assistance is particularly important. Nurses usually encounter intricate and swiftly evolving clinical situations, and learning is vital for safe and effective care [41]. Through help-seeking, nurses gain access to institutional knowledge, skilled co-workers, and practical strategies that enhance learning and competency [44]. Furthermore, in healthcare organizations, collective knowledge-building, promoted through help-seeking, is the key driver of learning [45]. Through help-seeking, nurses not only improve their control over task demands but also accrue intellectual and interpersonal resources, which enable enduring learning and adaptability [46]. Furthermore, seeking assistance can be observed as a resource investment behavior; it entails individuals consuming psychological resources (e.g. ego, vulnerability) to attain higher-value intellectual and interpersonal resources (e.g. mentorship, expertise).

In addition, prior empirical research validates this view. For instance, Sung and Thomas [47] found that help-seeking is positively related to goal achievement, signifying that individuals who actively request assistance are more likely to engage in exploratory and developmental learning. Likewise, Van der Rijt [48] and Chen et al. [49] noted that employees who often seek assistance from co-workers or supervisors report higher levels of on-the-job learning. This supports the notion that seeking assistance nurtures social learning by empowering individuals to access tacit knowledge, notice expert performance, and receive positive feedback. Moreover, seeking assistance accelerates individual learning and fosters team-level knowledge sharing, thereby facilitating a conducive learning environment [50]. In summary, a robust empirical and theoretical underpinning supports the notion that help-seeking behavior positively influences learning behaviors, especially in nursing contexts, where complexity and uncertainty require constant skill development. Therefore, we develop the following hypothesis:

#### H3

Help-seeking behavior is positively related to learning behavior.

### The sequential mediating mechanisms

The proposed sequential mediation conduit captures the dynamic process through which professional envy can act as a facilitator of constructive psychological and behavioral outcomes. This conduit is acutely embedded in COR theory [28, 46], which postulates that individuals are inspired to gain, hold, and defend important resources and that resource loss or threat activates compensatory behaviors aimed at resource gain. In the present framework, BPE signals a perceived resource gap when nurses compare themselves with more experienced or accepted co-workers [49]. Rather than culminating in dysfunctional or ineffective behaviors, envy may kindle a strategic resource-gaining process which is instigated by help-seeking behavior. Help-seeking acts as a prompt reaction to perceived resource inadequacy, facilitating employees access to external resources such as guidance, skills, knowledge, or emotional support, which are otherwise unobtainable internally [48].

This external resource attainment through seeking assistance is vital, as it offers foundational input for succeeding learning behaviors. Through help-seeking, nurses can engage in social learning processes, observational learning opportunities, and feedback mechanisms that excavate their professional capability [42]. Therefore, learning behavior embodies the vigorous utilization of attained resources and their conversion into superior competencies and work-related expertise [47]. This development from seeking assistance to learning behavior redirects a resource investment approach with COR theory, such as resources achieved from others being invested into learning, which then leads to future resource accretion. This is in line with the gain spiral models of engagement, where initial resource achievements support further resource generation, making a positive upward trajectory [46].

Subsequently, improved learning behavior among nurses fuels work engagement. Engaged nurses feel motivated and devoted to their work, facilitating higher performance and enhancing patient care [51]. This final outcome completes the resource gain chain, such that the early stimulating emotional state (envy) generates behaviors that restock and augment personal and job resources, resulting in persistent emotional and behavioral engagement. Moreover, prior research corroborates this sequence as an effective conduit in organizational context. For example, Monne et al. [42] and Chen et al. [49] noted that seeking assistance not only lessens doubt but also inspires profound learning and skill attainment, which, in turn, envisages greater engagement levels [52]. In conclusion, this sequential mediation conduit offers an empirically sound and theoretically supported explanation of how professional envy can initiate a positive chain reaction of behaviors that improve nurses work engagement. Therefore, we develop the following hypothesis:

#### H4

Help-seeking behavior and learning behavior sequentially mediated the relationship between benign professional envy and work engagement.

### The moderating role of inclusive leadership

From the lens of COR theory [28], BPE cues an apparent resource discrepancy that provokes nurses to pursue external resources through help seeking. However, transforming envy into seeking assistance requires a favorable working atmosphere because contextual factors either support or hinder resource investment. One such contextual enabler is inclusive leadership which plays a vital role in determining how nurses experience and react to multifaceted emotions such as envy [53]. Inclusive leaders create a culture in which employees feel valued and supported, thereby influencing their coping strategies in the presence of challenging emotions such as envy [54]. Such leaders create a supportive climate that inspires nurses to openly concede their developmental needs and strive for help without fear of judgment or negative concerns [20].

In addition, inclusive leadership augments the resource transformation process from seeking assistance to learning behavior. While seeking assistance offers access to psychological and instrumental resources, it does not always guarantee meaningful learning outcomes [55]. COR underscores that resource investments are most fruitful when entrenched in supportive corridors that permit resources to be stabilized and bundled [46]. By offering opportunities to practice, supporting feedback, and protecting nurses from punitive responses, inclusive leaders capitalize on the effectiveness of this adaptation, turning temporary help into robust proficiency [53]. This hustling effect not only reinforces the serial link between help-seeking and learning but also sets the stage for a greater gathering of personal resources, such as mastery and self-efficacy, which are core to fostering engagement.

Moreover, an inclusive environment facilitates and intensifies the transition process from learning to work engagement. Learning augments personal resources that nurture vigor, dedication, and absorption. However, inclusive leaders compound these gains by granting autonomy, recognizing progress, and creating opportunities for significant engagement [53]. Such actions accelerate the resource gain spiral, ensuring that recently assimilated proficiencies transform into enhanced roles rather than remaining underutilized [20, 53]. Together, these dynamics suggest that the indirect effect of BPE on work engagement via help-seeking and learning is conditional on inclusive leadership, such that the indirect effect is stronger when leaders actively create inclusive corridors. Conversely, in a low-inclusivity environment, BPE may fail to initiate help-seeking or produce nominal learning benefits, thereby weakening the sequential chain.

In addition, prior empirical research suggests that inclusive leadership positively influences psychological safety and trust [53, 54], which are crucial for seeking assistance behaviors. In addition, Khattak et al. [20] noted that such leadership nurtures relational identification, through which employees are associated with their leaders and inspired to engage in preemptive behaviors, such as help-seeking. Likewise, Nejati and Shafaei [54] found that inclusive leadership promotes learning behavior in organizations. Moreover, Wang et al. [55] found that such leadership fosters work engagement and innovative work behavior. Therefore, we develop the following hypothesis:

#### H5

Inclusive leadership moderates the indirect relationship between benign professional envy and work engagement through help-seeking and learning behaviors, such that the indirect relationship is stronger when inclusiveness is high, and vice versa.

## Methodology

### Sample and participants

Through cross-sectional survey approach, data from the study was collected during January 04, 2025 to March 10, 2025 from four major state-owned hospitals in Ankara. These hospitals typically operate with high nurse-to-bed ratios, reflecting the demanding workload and staffing pressures commonly observed in Turkish healthcare settings. According to the Organization for Economic Co-operation and Development (OECD, 2019), the general nurse-to-bed ratio in Turkish hospitals is 0.91 which is low compared to the OECD recommendation of 1.41 nurses per bed. A convenience sampling approach was used for data collection. The sample size was selected using the G*Power procedure with an effect size of 0.15, 90% power, and a significance level of 0.05. The minimum sample size recommended by G*Power was 169 participants. A 20% expansion was applied to account for potential sample loss, reaching a total sample size of 203. The inclusion criteria were as follows: (i) regular nurses in the selected hospitals, (ii) working in the same hospital for at least two years, and (iii) willing and unconditionally participate in the study. The exclusion criteria included (i) newly hired or fresh nurses with less than two years of experience in the same hospital and (ii) nurses hired temporarily or for a fixed period.

Before conducting the cross-sectional survey, we approached the hospital administration for formal approval. We discussed the study objectives with the hospital administration and heads of the nursing units. The nurses were assured of the anonymity and confidentiality of their responses. All ethical standards were strictly observed according to the Declaration of Helsinki and its amendments. Written and verbal consent for participation was obtained from the sampled nurses. We distributed 203 questionnaires to nurses and received 192 responses. Some of the surveys were either incomplete or incorrect; therefore, they were removed from the final dataset. The final sample comprised 183 responses, with a response rate of 90.1%.

### Ethical consideration

Before the formal survey, we obtained ethical approval from the Departmental Research Ethical Committee, Sinop University, Sinop, Turkiye, vide letter no. DoT-SU-24 dated 18/12/2024. We also approached the hospital administration for a formal approval. We discussed the study objectives and assured the confidentiality of the responses. The study objectives were also discussed with the concerned nursing staff, and it was assured that their responses would only be used for research purposes and their anonymity was highly prioritized. Moreover, we informed the participants that their participation in the study was voluntary and that they could withdraw at any stage of the study without reason.

### Measures

#### Benign professional envy (BPE)

BPE was assessed using 05 items scale developed by Lange and Crusius [31]. The sample item is “I strive to reach other people’s superior achievements.” We found good reliability (0.91) and CR (0.93) values for this scale.

#### Help-seeking behavior

Help-seeking behavior was measured through 03-item scale developed by Lee [56]. Through this scale, participants were asked to report the extent to which they approached various sources, such as supervisors, coworkers, and other departments, for help on a scale where 1 represented “rarely” and 5 represented “several times daily”. The sample item is “When I face difficulties in my nursing duties, I seek advice from my colleagues.” The Cronbach’s alpha and CR were 0.92 and 0.94 respectively.

#### Learning behavior

Learning behavior was measured using a seven-item scale developed by Edmondson [57]. This scale was contextualized in the Turkish nursing context. A sample item is “In our nursing team, we regularly seek feedback to improve patient care.” The Cronbach’s alpha and CR were 0.92 and 0.94 respectively.

#### Inclusive leadership

We utilized nine-item scale from Cameli et al. [52] to measure inclusive leadership. A sample item is “My leader is ready to listen to my request.” The Cronbach’s alpha and CR were 0.92 and 0.94 respectively.

*Work engagement*: Work engagement was assessed using a 09-item scale developed by Schaufeli et al. [32]. This scale measures work engagement in three dimensions: vigor, dedication, and absorption. The sample items were “At my job, I feel strong and vigorous”, “I am proud of the work I do”, and “I feel happy when I am working intensely.” The Cronbach’s alpha and CR were 0.92 and 0.94 respectively.

## Results

### Measurement model assessment

We employed a measurement model to assess scale reliability and validity. Reliability was confirmed using Cronbach’s alpha, rho_a, and composite reliability (rho_c). The acceptable range of reliability was 0.70 [58, 59]. Convergent validity was established using factor loadings and average variance extracted (AVE) values. The recommended threshold for factor loadings is that the loading values should be above 0.70 [59]. Similarly, the AVE value should be greater than 0.50. Collinearity was established using the variance inflation factor (VIF). The VIF value should be less than 5 to confirm no collinearity [58]. Discriminant validity was established using the Fornell and Larcker and Hetro-trait and Mono-trait (HTMT) ratios [60]. According to the Fornell and Larcker criteria, the square root of the AVE of a particular construct must be greater than the correlation of this construct with other constructs in the model. The HTMT matrix shows correlations that must not exceed the recommended threshold of 0.85 [61].

**Fig. 1:**
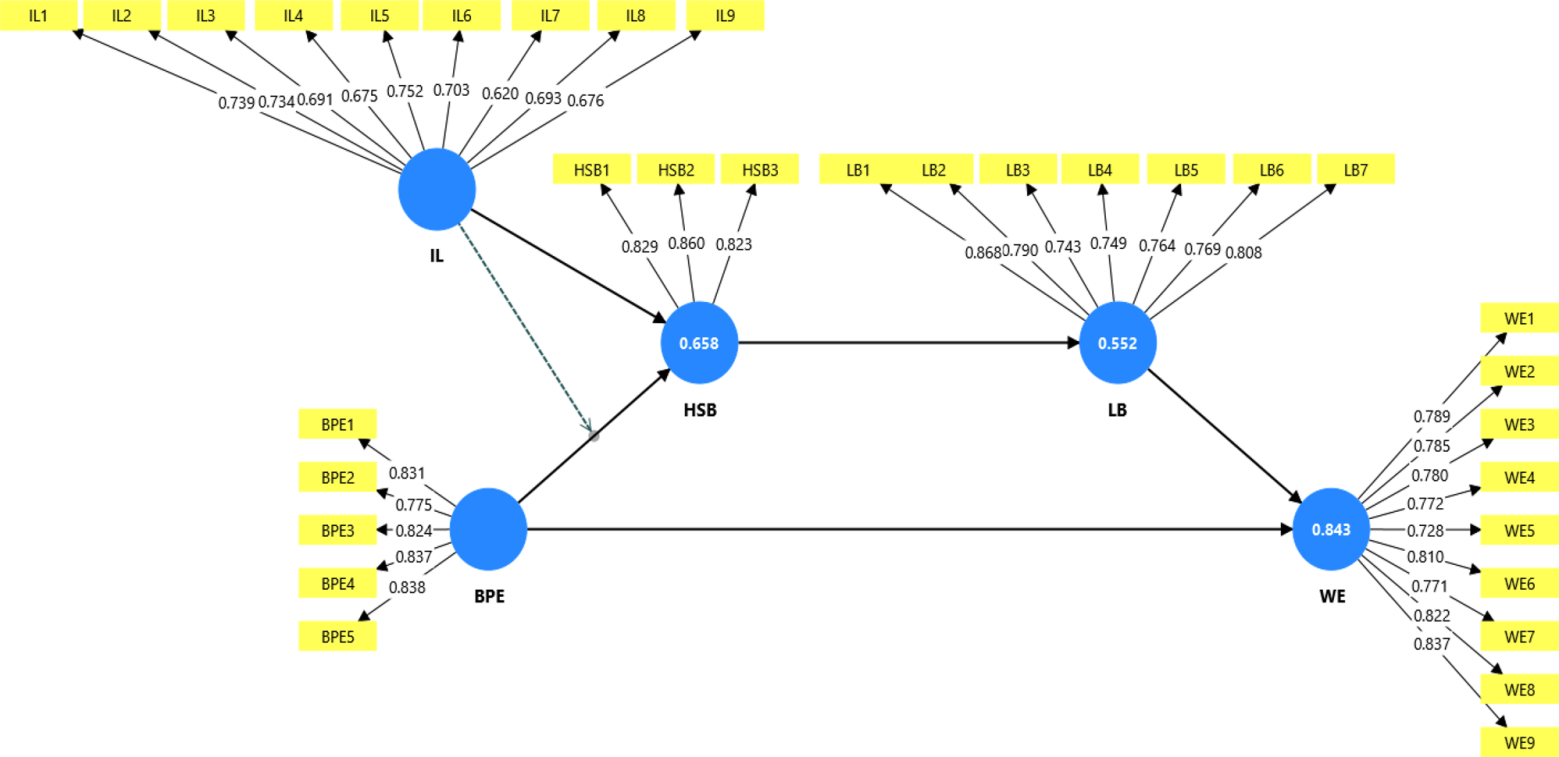
Measurement model assessment

Table 1 reports the factor loadings, VIF, Cronbach’s alpha, composite reliability (CR), and AVE. The factor loading values for all items of the constructs were above 0.70; however, some items had values lower than 0.70, but we retained these items as the overall CR and AVE values were above the suggested standards of 0.70 and 0.50, respectively [59–62]. The alpha and CR values were above 0.70; therefore, reliability was confirmed. The VIF values for all items were below 5, confirming no serious collinearity issues. Finally, the AVE values for all constructs were above 0.50; hence, convergent validity was established [58, 59]. In addition, the study collected data using a self-reported measure, and there might be a chance of common method bias (CMB). Hence, we assessed CMB using the variance inflation factor (VIF) values of the inner model. In the current study, all the inner model VIF values were lower than 3.33; therefore, the model can be considered free from common method bias [62]. The measurement model assessment is shown in Fig. 1.

**Table 1:** Reliability, convergent validity, and collinearity

Constructs	Items	Loadings	VIF	Cronbach’s alpha	Composite reliability (rho_c)	Average variance extracted (AVE)
Benign Professional Envy	BPE1	0.831	2.063	0.879	0.912	0.675
	BPE2	0.775	1.783			
	BPE3	0.824	2.068			
	BPE4	0.837	2.210			
	BPE5	0.838	2.242			
Help-seeking Behavior	HSB1	0.829	1.578	0.787	0.876	0.701
	HSB2	0.860	1.818			
	HSB3	0.823	1.620			
Inclusive Leadership	IL1	0.739	1.788	0.869	0.896	0.508
	IL2	0.734	1.739			
	IL3	0.791	1.615			
	IL4	0.675	1.554			
	IL5	0.752	1.906			
	IL6	0.703	1.629			
	IL7	0.720	1.473			
	IL8	0.693	1.661			
	IL9	0.776	1.606			
Learning Behavior	LB1	0.868	3.300	0.896	0.918	0.617
	LB2	0.790	1.993			
	LB3	0.743	1.726			
	LB4	0.749	1.834			
	LB5	0.764	1.857			
	LB6	0.769	1.927			
	LB7	0.808	2.633			
Work Engagement	WE1	0.789	2.239	0.924	0.937	0.622
	WE2	0.785	2.145			
	WE3	0.780	2.130			
	WE4	0.772	2.092			
	WE5	0.728	1.882			
	WE6	0.810	2.477			
	WE7	0.771	2.109			
	WE8	0.822	2.596			
	WE9	0.837	2.786			

Table 2 reports the Fornell and Larcker and HTMT matrix. The values of the square root of the AVE of all constructs (diagonal bold and italic) were higher than their correlations with other constructs; hence, discriminant validity was established. Similarly, the HTMT values (above the bold diagonal values) show that the correlations among the variables were less than 0.85; hence, we also established discriminant validity through this method.

**Table 2:** Fornell and larcker and HTMT

	BPE	HSB	IL	LB	WE
BPE	***0.821***	0.818	0.841	0.693	0.705
HSB	0.789	***0.837***	0.828	0.836	0.784
IL	0.790	0.732	***0.699***	0.687	0.721
LB	0.617	0.743	0.610	***0.786***	0.813
WE	0.638	0.670	0.650	0.713	***0.789***

Note: The bold italic values are the square root of the AVE. Below them are the correlations of the particular construct with other constructs. Above the bold italic diagonal values are the HTMT correlations matric

### Structural model assessment

After the measurement model, we employed the bootstrapping approach in SmartPLS 4 to calculate or check the structural path for the estimation of path coefficients (relationships among the study variables) and their statistical significance.

**Fig. 2:**
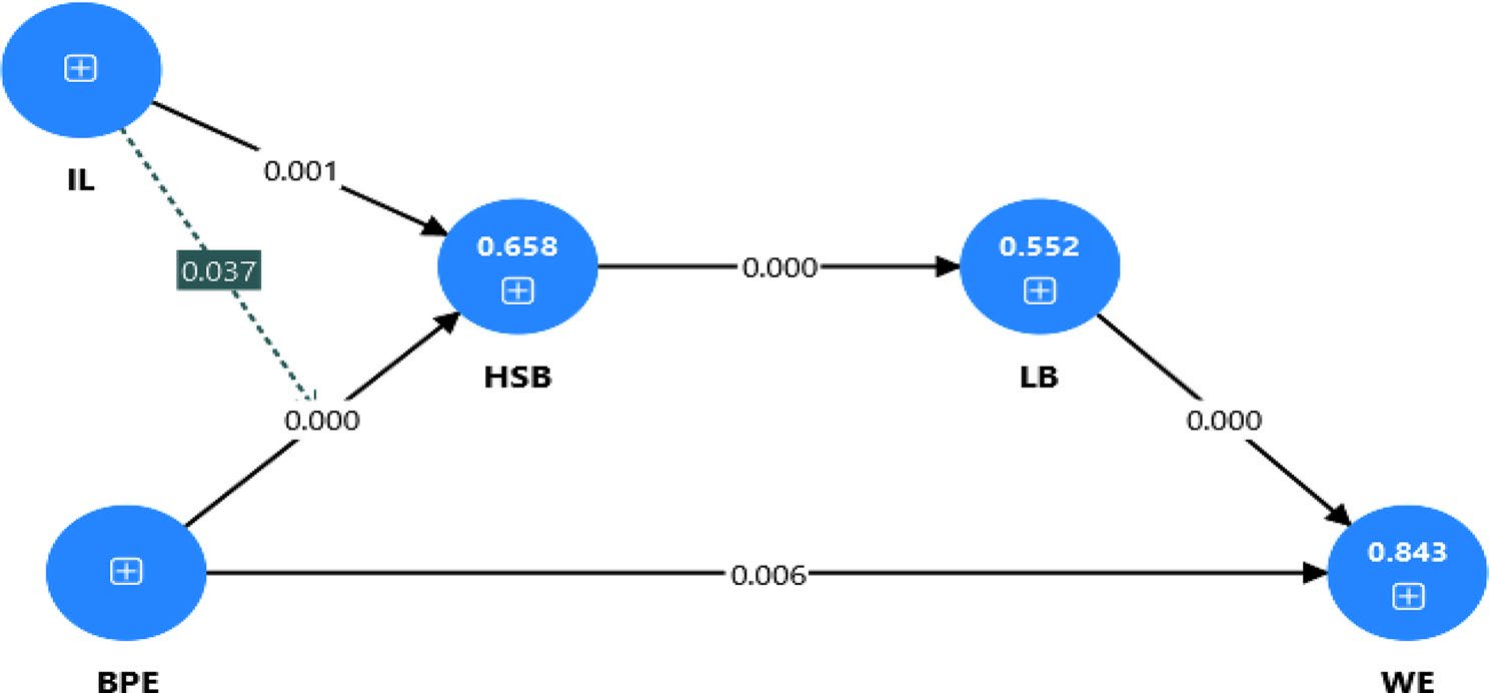
Structural model assessment

H1 assesses whether benign professional envy (BPE) significantly affects nurses’ work engagement. The results revealed that benign professional envy had a positive and significant impact on nurses’ work engagement (β = 0.120, t = 2.760, *p* < 0.05). Therefore, H1 is supported. Similarly, H2 evaluates whether benign professional envy significantly affects nurses’ help-seeking behavior. The findings suggest that BPE has a positive and significant effect on nurse help-seeking behavior (β = 0.558, t = 6.059, *p* < 0.001). Hence, H2 is supported. H3 evaluates whether help-seeking behavior (HSB) significantly affects nurses’ learning behavior (LB). The results revealed that HSB had a positive and significant impact on nurse learning behavior (β = 0.743, t = 15.263, *p* < 0.001). Finally, learning behavior was positively and significantly related to nurse work engagement (β = 0.839, t = 20.152, *p* < 0.001). The results are shown in Table 3. The structural model is shown in Fig. 2.

**Table 3:** Direct relationships

Hypotheses	Beta coefficient	Standard deviation	T statistics	*P* values
BPE -> WE	0.120	0.043	2.760	0.006
BPE -> HSB	0.558	0.092	6.059	0.000
HSB -> LB	0.743	0.049	15.263	0.000
LB -> WE	0.839	0.042	20.152	0.000

### Mediation analysis

A mediating analysis was performed to assess the mediating roles of help-seeking behavior (HSB) and learning behavior (LB) in the relationship between benign professional envy (BPE) and work engagement (WE). H4 evaluates whether HSB and LB sequentially mediate the relationship between BPE and work engagement. The results (see Table 4) revealed that the indirect effect of BPE on work engagement through HSB and LB was significant (β = 0.348, t = 5.538, *p* < 0.001). The total effect of BPE on work engagement was significant (β = 0.468, t = 7.157, *p* < 0.001), and the relationship between BPE and work engagement remained significant with the inclusion of mediators (β = 0.120, t = 2.760, *p* < 0.05). This finding highlights the complementary partial serial mediating roles of HSB and LB in the relationship between BPE and work engagement. Hence, H4 is supported.

**Table 4:** Mediation analysis

Total effect		Direct effect			Indirect effect of BPE on WE				Bootstrap 95% Conf. Int.		
BPE→WE		BPE→WE			H4: BPE→HSB→LB→WE						
B	t	*p*	B	t	*p*	B	se	t	*p*	Lower	Upper
0.468	7.157	0.000	0.120	2.760	0.006	0.348	0.063	5.538	0.001	0.243	0.447

### Moderation analysis

H5 evaluates whether IL moderates the indirect relationship between BPE and WE through HSB and LB. This study assessed the moderating role of inclusive leadership (IL) on the indirect effect of BPE on work engagement via HSB and LB. In the absence of a moderating effect (IL*BPE), the R-square value for work engagement was 0.623. This indicates that 62.3% of the change in work engagement is accounted for by BPE. With the introduction of the interaction effect, the R-square increased to 65.8%. This indicates a 3.5% increase in the variance explained by work engagement.

**Fig. 3:**
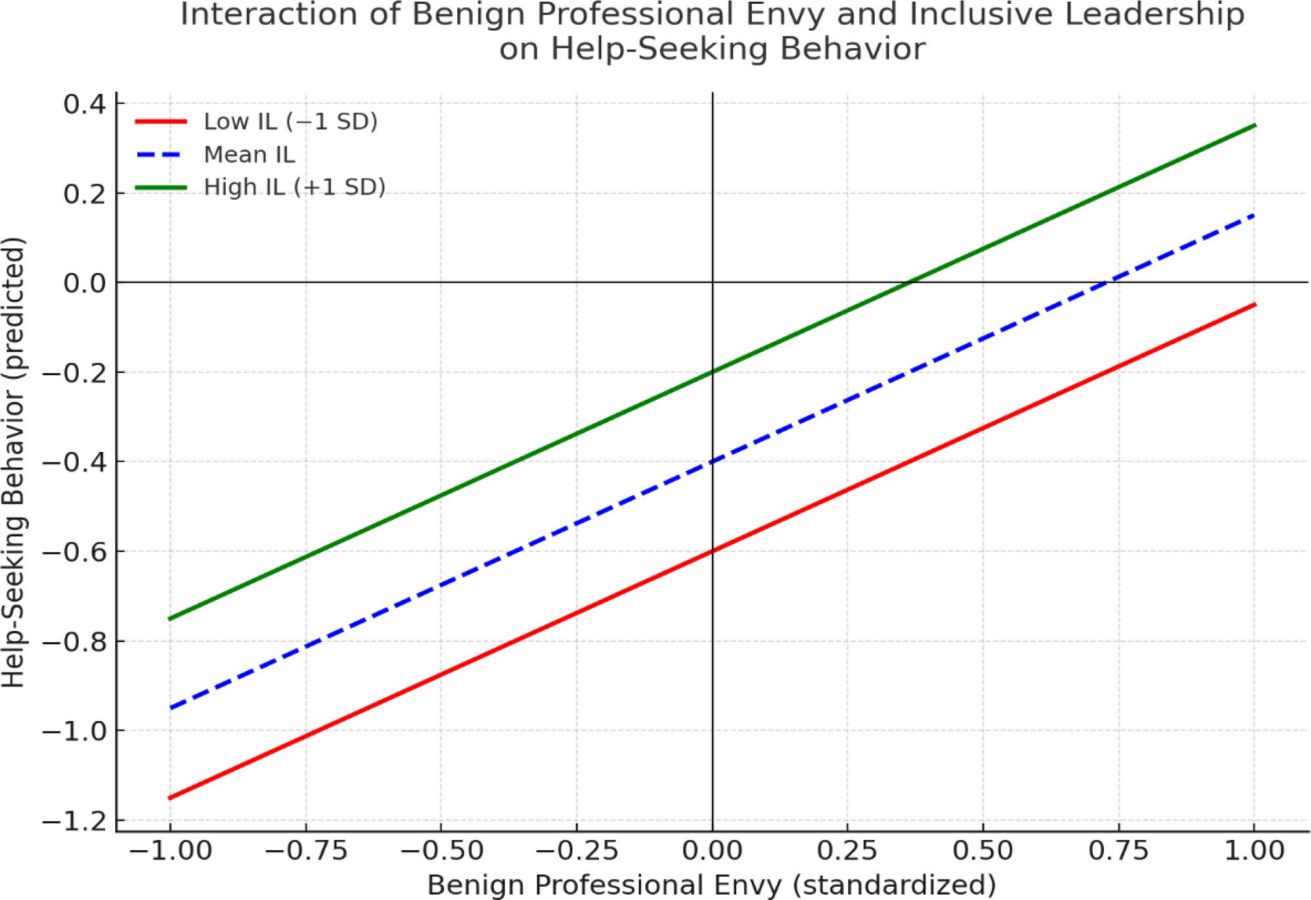
Simple slope analysis showing the moderating role of inclusive leadership (IL) on the relationship between benign professional envy (BPE) and help-seeking behavior (HSB). The positive association between BPE and HSB is stronger at higher levels of IL

The moderating effect was analyzed, and the findings showed a positive and significant effect of IL on the relationship between BPE and work engagement (β = 0.025, t = 2.099, *p* < 0.018), supporting H5. This indicates that with an increase in IL, the relationship between BPE and work engagement is stronger (see Table 5). Moreover, slope analysis was performed to better understand the nature of the moderating effect (see Fig. 3). As shown in Fig. 3, the line is much steeper at high IL levels, indicating that at high IL levels, the indirect relationship between BPE and work engagement via HSB and LB is stronger and vice versa. However, at low IL, the line tends to straighten, revealing that at lower IL, the increase in BPE does not lead to a similar change in HSB and subsequently work engagement.

**Table 5:** Moderation analysis

Hypotheses	Beta coefficient	Standard Error	T statistics	*P* values
H5:IL*BPE -> HSB -> LB -> WE	0.025	0.012	2.099	0.018

The f-square effect size was 0.011, which was extremely weak. Cohen [63] suggested that effect size of 0.02, 0.15, and 0.35 constitute small, medium, and large effect sizes respectively. In our case, there was a small or negligible moderating effect. This reveals that although the moderation is significant, it does not contribute much to explaining the dependent variable.

## Discussion

The results of this study offer significant insights into how envy can act as a positive motivational force in healthcare settings. Consistent with Conservation of Resources (COR) theory, the findings validate that nurses who experience BPE are more likely to organize and invest intrapersonal and interpersonal resources to improve their professional engagement. Instead of lessening energy, BPE stimulates nurses to enhance their capabilities, align with experienced peers, and commit more profoundly to their assigned tasks. This supports prior research signifying that BPE can nurture adaptive outcomes by inspiring self-development and favorable work behaviors [31, 37]. Within the resource-incentive environment of Turkish hospital care, where nurse-to-patient ratios are usually high and strain levels are generous, this proactive conclusion of BPE embodies a crucial emotional resource that can endure engagement.

The introduction of help-seeking behavior and learning behavior into the model and confirmation of its serial mediating role underscore the major contribution of this study. The findings indicate that nurses who experience BPE often engage in shared investigations and seek help from coworkers and peers. Such help-seeking behaviors not only reduce task-related dubiety but also raise opportunities for vicarious learning. For instance, the transformation of help-seeking into active learning behaviors might enable nurses to amass new knowledge, polish their skills, and improve their sense of mastery, leading to fortifying their engagement. This serial passageway is particularly evocative in nursing, where patient safety, clinical decision-making, and care quality rely on persistent learning and teamwork. Our results are consistent with previous research, which stressed that pliable responses to workplace envy can simplify knowledge attainment and skill improvement, thus nurturing constructive organizational outcomes [37].

Notably, the moderating role of inclusive leadership was established, elucidating that inclusive leaders magnify the favorable effects of BPE on work engagement through the intervening mechanisms of help-seeking and learning behavior. However, our findings suggest a significant but weak moderating effect of inclusive leadership. This specifies that, while theoretically important, the moderating role has limited practical significance, signifying that inclusive leadership alone may not produce considerable changes in the envy-engagement link without other supportive factors. Other contextual resources, such as organizational culture, workload management, and structural support, may overshadow or interact with leadership behaviors. For practice, this means leadership interventions should be embedded in a broader framework of organizational policies (e.g., participatory decision-making, flexible scheduling, and team-based learning opportunities) to create conditions where inclusive leadership can produce more meaningful gains. Leaders blended with inclusive characteristics such as openness, accessibility, and appreciation create a psychologically safe atmosphere where nurses feel contented confessing limitations and reaching out for assistance without fear of reprisal. This inclusiveness diminishes social hurdles to help-seeking and strengthens a culture of collective learning, thus triggering the conversion of BPE into personal development and engagement. In the Turkish nursing context, where workload compressions and hierarchical structures may otherwise dampen open dialogue, inclusive leadership serves as a crucial contextual enabler that accelerates the effective utilization of personal and social resources. This result supports COR theory’s contention that resource-rich climates facilitate employees’ better investment in and defense of their resources [47]. Taken together, these results expand the theoretical understanding of envy in organizational contexts by demonstrating that its influence is dependent on resource investment mechanisms and contextual factors such as leadership.

### Theoretical implications

The present framework offers several important theoretical contributions by exploring how BPE operates in healthcare settings. The finding that BPE significantly improves nurses’ work engagement validates that envy, usually characterized as a caustic emotion, can serve as a favorable motivational resource when managed constructively. This study extends the COR perspective by demonstrating that emotions can serve as instigating resources that activate consequent investment behaviors. Precisely, envy motivated nurses to seek assistance from coworkers, and through this help-seeking, they attained new skills and knowledge that reinforced their learning behaviors. This serial passageway confirms that BPE can prompt a resource-gain spiral, in which the early emotional resource leads to collective resource enlistment (help-seeking), which further improves into intellectual resources (learning behavior), eventually concluding in higher engagement.

Additionally, the important role of inclusive leadership as a moderator underscores that the transformation of envy into favorable outcomes is not automatic but relies on contextual factors. Such leaders provide a psychologically safe working environment where nurses can freely express help-seeking intentions without any fear, thereby intensifying the indirect conduit from envy to work engagement. This finding contributes to the leadership literature by strengthening the notion that leadership behaviors act as vital boundary conditions in resource processes. It also nuances COR theory by showing that contextual resources (inclusive leadership) do not simply shield resource loss but energetically intensify resource gains.

Lastly, this research was conducted in the Turkish nursing context, where nurse-to-bed ratios are comparatively higher than the international standard and hierarchical structures still dictate, the results disclose that even in resource-shortage climates, BPE can be transmuted into engagement through social and learning mechanisms. This contextual extension highlights that psychological and leadership forces work differently across cultures and professions, widening the theoretical applicability of COR theory and envy research in this context.

### Managerial implications for nursing

This study suggests several important implications for nursing managers and hospital administration. First, the confirmation that PE improves work engagement by seeking assistance and learning signifies that envy should not be repressed but positively managed. Healthcare management should cultivate interventions that stabilize envy as a driver of development. For example, knowledge-sharing workshops and peer gratitude strategies can inspire nurses to turn upward social comparisons into dynamic learning opportunities.

Second, the serial intervening chain underscores that help-seeking behavior is an essential first step. However, in many nursing environments, seeking assistance may be stigmatized as a sign of infectivity. The results underscore the significance of reframing help-seeking as a professional strength. Healthcare organizations can institute clinical supervision systems, mentorship programs, and peer-support groups to encourage a culture where asking for professional or work-related help is regarded as a way to enhance capability rather than a sign of weakness.

Third, the positive and significant impact of learning behavior in the mediating conduit indicates that engagement is not merely psychological but also skill-based. Healthcare leaders should offer organized opportunities for constant professional learning, such as simulation-based training, inter-and intradepartmental knowledge exchange, and meditative practice sessions. This confirms that the dynamism engendered by BPE is routed into assessable skill improvement, which endures engagement over time.

Finally, the important role of contextual enablers, such as inclusive leadership, calls for leadership development initiatives that explicitly target inclusivity. Unfortunately, in Turkish healthcare systems, leaders frequently work under hierarchical structures which may depress open communication. Therefore, special training programs for supervisors and head nurses should be arranged that specifically emphasize inclusive practices, such as valuing contributions irrespective of seniority, welcoming novel and diverse opinions, and reacting compassionately to help-seeking requests. In this way, leaders can transmute delicate emotions into favorable outcomes that benefit both staff and patients.

### Limitations and future directions

This study had some limitations. First, we tested the proposed moderated sequential mediation framework using cross-sectional data, which confines us to draw causal conclusions. However our findings support the notion of a resource-based gain spiral consistent with COR theory, the cross-sectional design restricts us from establishing causal direction. The observed relationships should therefore be interpreted as associations. Future longitudinal or experimental research is essential to verify whether benign envy truly initiates a gain spiral over time. Moreover, multi-source data, for example, peer or supervisor assessments of help-seeking and engagement, would further strengthen causal inference and reduce common method variance, offering a more rigorous test of the resource-accumulation mechanism proposed by Conservation of Resources theory. Second, the study depended on single-sourced and self-reported data, which may lead to social desirability issues, particularly given the sensitivity of emotions such as envy and help-seeking in professional settings. In future, researchers can choose multi-waves (different time intervals) and multi-source data, that is, peer or supervisor evaluations, to triangulate findings. Third, we studied only benign envy and ignored malicious envy. Investigating how inclusive leadership and resource dynamics form the contrary impacts of benign and malicious envy would offer a complete picture. Fourth, although this research accentuates work engagement as an outcome. Future research could extend our model by adding other performance-related outcomes, such as teamwork effectiveness, patient safety, and innovation in clinical practice. Finally, this study was conducted in Turkish healthcare settings, where organizational and cultural norms underscore authority and the hierarchy. This situation may have augmented the moderating effect of the inclusive leadership. These cultural and structural conditions may limit the extent to which the observed relationships, particularly the role of benign envy and leadership, apply to contexts with flatter hierarchies or more balanced nurse–patient ratios. Future research could replicate the study model across countries or clinical settings with different cultural orientations (e.g. hierarchical vs. egalitarian, individualist vs. collectivist) to assess its generalizability.

## Conclusion

The present research develops an understanding of how an apparently negative emotion, such as envy, can act as a positive dynamic in nursing. By establishing that BPE improves work engagement through the serial mechanisms of help-seeking and learning and that inclusive leadership intensifies this route, the results provide both theoretical enhancement and practical significance. Within the Turkish nursing setting, where nurses usually work under hierarchical structures and high patient loads, the findings underscore the significance of nurturing inclusive leadership and supportive professional cultures that facilitate assistance-seeking and constant learning. Finally, this study illustrates that when constructively handled, envy can be transferred into a valued resource that reinforces engagement, enhances professional development, and contributes to the sustainability of healthcare organizations.

## Data Availability

The data and materials related to the manuscript is available from the corresponding author upon reasonable request.

## Abbreviations

COR: Conservation of Resources
BPE: Benign Professional Envy
HSB: Help-seeking Behavior
LB: Learning Behavior
IL: Inclusive Leadership
WE: Work Engagement
SD: Standard Deviation
HTMT: Hetro-trait and Mono-trait
AVE: Average variance extracted
VIF: Variance inflationary factor
CR: Composite reliability
